# Insulin Promoter Factor 1 variation is associated with type 2 diabetes in African Americans

**DOI:** 10.1186/1471-2350-6-37

**Published:** 2005-10-17

**Authors:** Mohammad A Karim, Xiaoqin Wang, Terri C Hale, Steven C Elbein

**Affiliations:** 1 Endocrinology Section, Medical Service, Central Arkansas Veterans Healthcare System, College of Medicine, University of Arkansas for Medical Sciences, Little Rock, Arkansas 72205 USA; 2 Division of Endocrinology and Metabolism, Department of Internal Medicine, College of Medicine, University of Arkansas for Medical Sciences, Little Rock, Arkansas 72205 USA

## Abstract

**Background:**

Defective insulin secretion is a key defect in the pathogenesis of type 2 diabetes (T2DM). The β-cell specific transcription factor, insulin promoter factor 1 gene (*IPF1*), is essential to pancreatic development and the maintenance of β-cell mass. We hypothesized that regulatory or coding variants in *IPF1 *contribute to defective insulin secretion and thus T2DM.

**Methods:**

We screened 71 Caucasian and 69 African American individuals for genetic variants in the promoter region, three highly conserved upstream regulatory sequences (PH1, PH2 and PH3), the human β-cell specific enhancer, and the two exons with adjacent introns. We tested for an association of each variant with T2DM Caucasians (192 cases and 192 controls) and African Americans (341 cases and 186 controls).

**Results:**

We identified 8 variants in the two populations, including a 3 bp insertion in exon 2 (InsCCG243) in African Americans that resulted in an in-frame proline insertion in the transactivation domain. No variant was associated with T2DM in Caucasians, but polymorphisms at -3766 in the human β-cell enhancer, at -2877 bp in the PH1 domain, and at -108 bp in the promoter region were associated with T2DM in African American subjects (p < 0.01), both individually and as haplotypes (p = 0.01 correcting by permutation test). No SNP altered a binding site for the expected β-cell transcription factors. The rare alleles of InsCCG243 in exon 2 showed a trend to over-representation among African American diabetic subjects (p < 0.1), but this trend was not significant on permutation test.

**Conculsion:**

The common alleles of regulatory variants in the 5' enhancer and promoter regions of the *IPF1 *gene increase susceptibility to type 2 diabetes among African American individuals, likely as a result of gene-gene or gene-environment interactions. In contrast, *IPF1 *is not a cause of type 2 diabetes in Caucasians. A previously described InsCCG243 variant may contribute to diabetes susceptibility in African American individuals, but is of low penetrance.

## Background

Type 2 diabetes (T2DM) has a substantial genetic component, but genetic heterogeneity, gene-environment interactions, and a large number of loci with small effect have combined to confound the identification of susceptibility genes. The pathogenesis of T2DM is characterized by early resistance to insulin-mediated glucose uptake and β-cell dysfunction followed by the further inexorable decline in function and possibly mass [1]. To maintain insulin secretion and glucose homeostasis in the face of resistance to insulin mediated glucose uptake, the β-cell must increase insulin secretion, either by increased function or increased β-cell mass, a concept known as β-cell compensation [2]. Impaired β-cell function predicts future diabetes [3], and work from our laboratory [4] and others [5,6] suggest that the ability of the pancreatic β-cell to compensate for prevailing insulin sensitivity (ie, β-cell compensation) is highly heritable. Nonetheless, the genetic controls over β-cell failure are largely unknown.

The islet transcription factor, insulin promoter factor 1 (*IPF1*) gene (also known as the pancreatic duodenal homeobox 1, *PDX1*, and insulin upstream factor 1, *IUF1*), is required for both the differentiation and maintenance of the β-cell phenotype [7]. The importance of *IPF1 *in pancreatic β-cell development and function is demonstrated in naturally occurring human mutations and in experimental mouse models. Humans lacking a functional *IPF1 *allele have pancreatic agenesis [8], whereas humans heterozygous for the same variant develop early onset, insulin deficient diabetes (Maturity Onset Diabetes of the Young, MODY4) [9]. Similarly, mice homozygous for targeted disruption of *IPF1 *(*PDX1*) fail to develop a pancreas, whereas haploinsufficient mice have impaired glucose-stimulated insulin secretion and develop T2DM with aging [7]. Impaired glucose homeostasis with reduced *IPF1 *activity likely derives from an influence on both β-cell mass and function. Both isolated mouse islets and dispersed β-cells with haploinsufficiency for *IPF1 *showed increased apoptosis even at basal glucose levels, but were functionally normal [10]. However, IPF1 also transactivates the promoters of multiple islet-specific genes, including insulin, the GLUT2 islet glucose transporter, islet amyloid polypeptide (IAPP), and somatostatin [11]. Thus, *IPF1 *sequence variants might be expected to influence both β-cell mass and the expression of key β-cell genes.

Regulation of *IPF1 *gene expression is complex, with multiple upstream regulatory elements that bind other key β-cell genes including HNF3β, HNF1α, and SP transcription factors [12,13]. Upstream sequences include a human and β-cell specific enhancer region at -3.7 kb to -3.4 kb that binds HNF1α, HNF3β, SP1, and SP3, and 3 additional regions that are highly conserved between mouse and human: PH1 (-2.6 kb to -2.8 kb), PH2 (-2.1 kb to -2.2 kb), and PH3 (-1.6 kb to -1.9 kb) [14]. PH1 and PH2 bind HNF3β, and PH1 also binds IPF1 [12] and HNF1α [15].

Multiple studies have examined the *IPF1 *gene for mutations in early onset, autosomal dominant diabetes and a few studies have searched for mutations in T2DM in Caucasians [16-19]. Only rare coding variants have been identified. However, neither the far upstream regulatory regions nor other ethnic groups including African Americans have been examined. We hypothesized that common variants in coding or regulatory regions of the *IPF1 *gene contribute to the failure of β-cell compensation and to the susceptibility to common T2DM. To address this hypothesis, we screened the coding and upstream regulatory regions of the *IPF1 *gene in both African American and Caucasian diabetic individuals with a family history of diabetes. We then tested both individual variants and haplotypes for diabetes susceptibility using a case-control design for each population.

## Methods

### Experimental subjects

We examined two populations: Caucasian individuals ascertained primarily for Northern European Ancestry, and African American individuals. Screening for new sequence variants was conducted in two stages. Initial studies of coding, 5' and 3' untranslated regions, 5' flanking region, and far upstream enhancer and regulatory elements upstream were conducted in 48 individuals: 12 African American subjects with T2DM, 12 African American control subjects, 12 Caucasian subjects with T2DM, and 12 nondiabetic Caucasian subjects. To better detect uncommon coding variants, we subsequently screened an additional 45 African American subjects with T2DM and an additional 47 Caucasian subjects with T2DM for exonic regions. Thus, exons were screened in total for 12 African American control subjects and 57 African American subjects with T2DM, and for 12 Caucasian control subjects and 59 Caucasian subjects with T2DM. To further improve sensitivity to detect coding variants, we selected affected subjects with early onset of T2DM: ages 25 – 40 years in African American subjects, and ages 30 – 45 years in Caucasian subjects.

Case control studies were conducted similarly in both Caucasian and African American populations. Our Caucasian study comprised 188 unrelated nondiabetic control individuals (73 male, 115 female) and 190 individuals with T2DM (133 male, 57 female), and has been described previously [20]. This population has 80% power to detect an absolute difference in allele frequencies of 10% difference between cases and controls for minor allele frequencies in controls of 10% to 50%. Initial case-control studies in African Americans were conducted on 165 control individuals (82 male, 83 female) and 255 diabetic cases (142 male, 113 female). This population likewise has at least 80% power to detect a difference between case and control allele frequencies of 10% over the range of allele frequencies from 10% to 50%. For both case-control studies, all diabetic individuals had at least one diabetic first degree relative. Control individuals had a normal 75 g oral glucose tolerance test or a fasting or random glucose below 5.6 mmol/l, and no diabetic first degree relative. No individual with known impaired glucose tolerance was included in either group, but because some subjects were ascertained at health fairs, not all subjects underwent glucose tolerance testing and thus impaired glucose tolerance could not be excluded.

During the course of this study, additional African American samples became available. Given the evidence for association and newly available samples, we subsequently expanded typing for SNPs 1, 4, 11, and the INSCCG243 variant by an additional 21 African American cases and 85 African American controls. Hence, for these markers we present data for on 186 African American controls (95 male, 91 female) and 341 African American diabetic individuals (186 male, 155 female). The African American control population had a BMI of 30.2 ± 7.1 kg/m^2 ^and an age of 42.7 ± 13.0 years. The African American T2DM population had a BMI of 32.4 ± 7.3 kg/m^2^, and age of diabetes diagnosis of 42.6 ± 11.9 years, and an age at testing of 55.0 ± 12.6 years. All subjects provided informed consent under protocols approved by the University of Utah or University of Arkansas for Medical Sciences Institutional Review Boards.

### Mutation detection and genotyping

**Figure 1:**
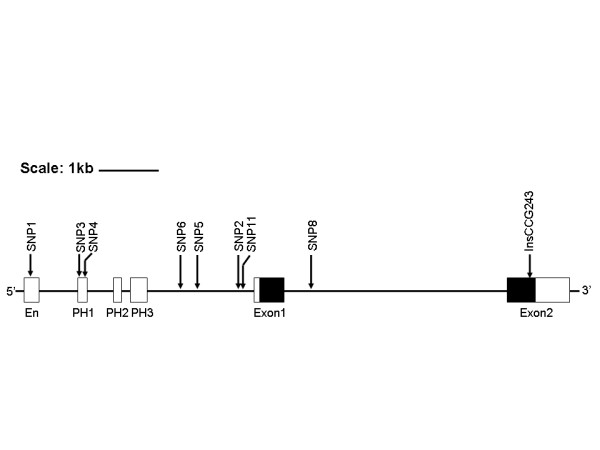
Map of the IFP1 gene and upstream regulatory regions. Upstream regulatory regions are shown in white boxes. Exons are shown in boxes; translated regions are black and the untranslated regions are grey. The approximate locations of each SNP and the CCG (proline) insertion polymorphism are shown. Lines with arrow heads show the areas covered for PCR/DHPLC screening.

We designed primers from the human genome sequence (AL353195) and alignment with the human *IPF1 *mRNA sequence (NM_000209) to cover exons 1 and 2, the 5' and 3' untranslated regions, 1.5 kb of 5' flanking and proximal promoter sequence, and the reported enhancer and regulatory elements PH1, PH2, and PH3 at positions -3.6 kb, -2.76 kb, -2.2 kb, and -1.76 kb from the ATG start site [12,15] (Figure 1). Initial screening was by denaturing high pressure liquid chromatography (DHPLC) using a Transgenomic WAVE HT DNA Fragment Analysis System (Transgenomic, Inc, Omaha, NE). Altered migration was confirmed and characterized by bidirectional sequence analysis [21] using infrared dye-labeled primers and GR4200 Sequencers (LI-COR Biotech, Lincoln, NE).

The proline insertion variant (InsCCG243) was typed using infrared dyes with detection on a LICOR GR4200 sequencer and scored using SAGA ^GT ^fragment analysis software (LICOR Biotech). Because the sequences in the exon 2 around the InsCCG243 variant are highly G-C rich, we confirmed our results using the Advantage GC-2 PCR kit (BD Biosciences Clontech, Palo Alto, CA). The remaining 8 SNPs were genotyped by Pyrosequencing on a PSQ-96 machine according to manufacturer methods (Biotage AB, Uppsula, Sweden). Primer sequences are available in Table 1.

### Statistical and binding factor analyses

Our primary analysis was allelic association. Allelic frequencies in cases and controls were compared using the Fisher exact test. We report both the uncorrected p values for allelic association and the simulated p values which correct for the number of tests using HaploView version 3.2 [22]. We considered p < 0.05 to be significant without correction for multiple testing. In exploratory analyses, we also examined SNPs with an allelic association of p < 0.10 using several analyses. First, we tested for a genotypic association using the Fisher Exact Test under dominant and recessive models. Second, we tested for association using logistic regression analysis under additive, dominant and recessive models. For uncommon SNPs in which few recessive individuals were observed, recessive and additive models were not tested. Logistic regression included age (testing age for controls, age of diagnosis for cases), ln-transformed body mass index (BMI), and gender as covariates.

Pair-wise linkage disequilibrium coefficients were calculated by allele counting from the combined case and control data. Phase was estimated using the Expectation Maximum algorithm. Haplotype distribution between cases and controls was tested using Phase v2.1.1 [23], Arlequin [24], or HaploView v3.2 [22]. TagSNPs were selected using the LDSelect program based on the correlation between SNPs (r^2^) set at 0.8 [25]. Altered transcription factor binding sites were identified using the TFSEARCH program based on the TRANSFAC database [26].

## Results

We detected a total of 9 sequence variants, including 8 SNPs in noncoding regions and a single coding variant observed only in African American subjects and comprising a 3 bp CCG/proline insertion (InsCCG243) in exon 2 (Table 2 and Figure 1). No other common or rare coding variants were detected among a total of 138 African American or 142 Caucasian alleles, including the previously reported D76N variant [17,19]. We identified 3 SNPs in the far 5' regulatory sequences, including one SNP in the human-specific enhancer region (SNP1) and two SNPs (SNP3, SNP4) in the PH1 region. SNP3 was common among Caucasians but not observed in African Americans. Two additional SNPs (SNP2, SNP11) were located in the proximal 5' flanking region (Figure 1).

We tested each of 7 SNPs that had minor allele frequencies over 10% in 190 Caucasian individuals with T2DM and 188 Caucasian control individuals. No individual SNP was associated with T2DM (p > 0.9 on permutation p value for all; Table 2 shows allelic frequencies, Table 3 provides raw numbers). The complete variation in the 7 SNPs could be captured with only 4 tagSNPs (SNPs 1, 2, 3, and 4 at positions -3766, -2890, -2877, and -279). All 7 SNPs fell into a single haplotype block with pairwise D' values of 1.0. Consistent with these observation, only 4 haplotypes were observed at over 1% frequency (Table 4). The 4 haplotypes could be distinguished by typing SNP 2, SNP 3, and SNP 4. Neither the distribution of haplotypes in Caucasians (p = 0.98 by Phase v2.1.1), nor any individual haplotype (p > 0.44; Table 4) was associated with T2DM.

**Figure 2:**
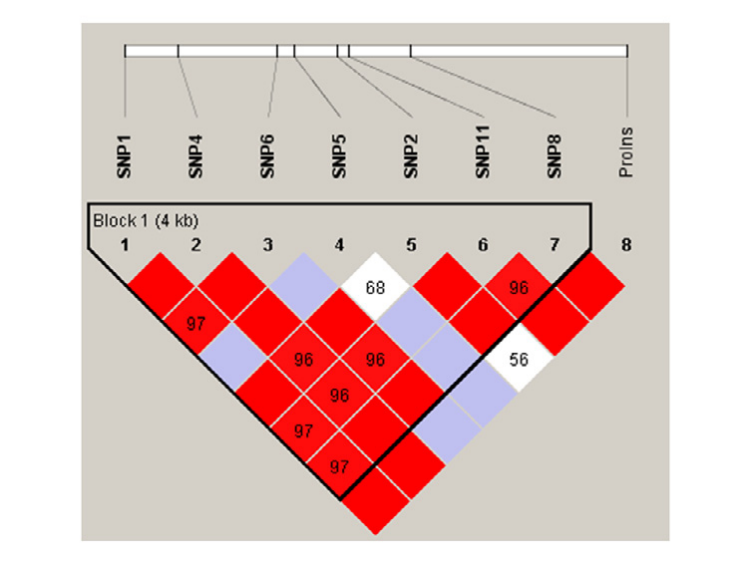
Linkage disequilibrium (D') plot of the IPF1 gene in African American subjects. SNPs are shown by location as in Table 1 and Figure 1. Squares without numbers represent D' values of 1.0; all numbers represent the D' value expressed as a percentile. Red squares represent pairs with LOD score for linkage disequilibrium of ≥ 2, blue squares represent D' = 1 but LOD<2, and white squares represent LOD<2 and D'<1.0. Plots were generated using HaploView v3.2.

In contrast to Caucasians, SNP1 in the human β-cell specific enhancer, SNP4 in the PH1 region, and SNP11, a G insertion in the proximal 5' flanking region, were significantly associated with T2DM (p = 0.007, p = 0.008, and p = 0.0008, respectively), with predicted odds ratios for the major allele of 1.59, 1.45, and 1.79, respectively. In contrast, the proline insertion in exon 2 (InsCCG243) showed a trend to an association, but did not reach statistical significance even without correction for multiple testing (p = 0.088, OR 1.58). The common alleles of SNPs 1, 4, and 11 were over-represented in subjects with T2DM, whereas the insertion allele of InsCCG243 was increased in T2DM subjects. SNPs 1 and 11 were in strong linkage disequilibrium (r^2 ^= 0.927). Based on r^2^>0.8, we could capture the full diversity among African American subjects with 6 tagSNPs: SNPs 1, 2, 4, 5, 6, and InsCCG243. Using all observed variants, we identified only 7 haplotypes with over 1% frequency. Only Ins243CCG fell outside of the block defined using confidence interval definitions (Figure 2). Although the overall distribution of haplotypes was different between cases and controls (permuted p = 0.01), no single haplotype was over-represented in cases compared with controls (Table 5). In contrast, when the 3 individually associated variants were examined together, 2/3 haplotypes showed 8% differences between cases and controls (Table 5). The Ins243CCG proline insertion split the most common haplotype, and occurred on a single haplotype that showed a similar distribution between cases and controls as the Ins243CCG SNP (Table 5). When Ins243CCG was included in the analysis, neither major haplotype (CAID or CAII, where I is the insertion of the G at SNP 11 or the proline at Ins243CCG and D is the absence of the extra bases) was associated with T2DM when the proline insertion was included, but was associated when not split. Hence, the proline insertion was not driving the observed association.

In exploratory analyses, we sought to determine the most likely mode of inheritance and to determine whether the observed allelic associations were modulated by age, age of onset, or obesity. SNPs 1 and 11 acted as a recessive trait for the major allele (p = 0.017 and 0.003, respectively), whereas SNP 4 acted as a dominant trait for the major allele (p = 0.0016). No other SNP was associated with T2DM on exploratory analyses. Logistic regression confirmed the allelic association tests. Only SNPs 1, 4, and 11 and BMI were significant factors in the model. SNP 4 showed a stronger dominant effect (p = 0.0002, OR 3.23) with correction for age, BMI, and gender, whereas SNPs 1 and 11 were again consistent with a recessive effect of the major allele (p = 0.017 and 0.003, respectively, and OR 1.713 and 1.916, respectively).

The haplotype analysis and association analyses did not suggest which of the 3 SNPs was driving the observed association. No SNP altered the binding sites for known β-cell regulatory factors, including HNF1α, HNF1β, HNF3β, SP1/3, or auto-regulatory IPF1/PDX1 binding [13]. However, the minor allele of SNP1 abolished the predicted binding of heat shock factors 1 and 2 (HSF1 and HSF2), which are involved in cellular stress responses [27].

## Discussion

As a key transcription factor in the pathways controlling both β-cell mass and essential genes for insulin biosynthesis and secretion, *IPF1 *is a strong candidate for the inherited defect in insulin secretion that characterizes T2DM and the prediabetic state. Mutations in *IPF1 *are a rare cause of early onset T2DM (MODY4)[16,18,28]. Several previous studies have searched for mutations in late onset T2DM among Caucasians [17,19,28] with variable results, but these studies have not focused on the well described conserved elements that extend 5 kb upstream of the ATG translation start site. Furthermore, no published study has examined a non-Caucasian population. The role of previously reported, rare nonsynonymous SNPs in typical T2DM [17,19] is unclear. We recently were unable to demonstrate a major role in T2DM susceptibility or in reduced insulin secretion for the most common of these missense variants, D76N, among Caucasians [29]. In screening 282 African American diabetic and 96 African American control subjects, we observed the D76N variant only in 3 individuals with T2DM (allelic frequency 0.005), and thus lacked the power to evaluate this variant in African American subjects. In the present study, we found no new coding variants among Caucasian samples, nor were any of 6 SNPs in the 5' flanking region, including those in the enhancer and PH1 domains, associated with T2DM in Caucasians. The lack of involvement of *IPF1 *in Caucasians was supported by the haplotype analysis.

In contrast, 3 of 8 sequence variants in African Americans were associated with T2DM, and two variants that were not seen in Caucasians showed a trend to an association. SNPs 1 and 11 (G insertion at -108 bp) were in strong linkage disequilibrium. SNP11 was previously reported in Japanese, where the (G)_4 _allele was less common than in African Americans and was of similar frequency in 88 cases and 67 controls [30]. Genetic studies likely cannot distinguish the impact of SNP1 in the enhancer and SNP11 in the proximal promoter on *IPF1 *transcription. The association of SNP11 was statistically the strongest. The haplotypes constructed from SNPs 1, 4, and 11 together confirmed the individual SNP results, but the association was not stronger using either PHASE or HaploView 3.2 than observed for individual SNPs. Hence, we cannot determine whether SNPs 1, 4, and 11 might interact to increase the risk of T2DM. Examination of the two haplotypes that were associated with T2DM showed that the risk and protective haplotypes differed at all three positions (CAG_4 _vs TTG_3_). Only the G_3 _allele uniquely distinguished a haplotype that differed in frequency between cases and controls. Notably, this haplotype is protective with regard to T2DM susceptibility. The "risk" haplotype (GAG_4_) is very common (71% of T2DM, 62.5% of controls). The contribution of the high prevalence allele to T2DM susceptibility has also been seen in other T2DM susceptibility genes, such as the PPARγ Pro12Ala variant [31].

The SNPs detected in this study are not predicted to alter the binding of known regulators of *IPF1 *gene expression, including HNF1α, HNF3β, or IFP1/PDX1, which have been shown to bind to these two regions [14]. SNPs 3 and 4 lie only 33 bp apart in the highly conserved PH1 element and approximately 50 bp upstream of the binding sites for known β-cell transcription factors NKX2.2, PBX1, and HNF3β. Several predicted binding sites for other transcription factors are altered by these associated variants. As noted above, the minor (T) allele of SNP1 is predicted to abolish the binding of heat shock proteins HSF1 and HSF2, although the role in insulin secretion or β-cell function is speculative. The common (A), T2DM-associated allele of SNP4 was predicted to abolish binding of the transcription factor Ets1. Ets1 has been described primarily in oncogenesis and angiogenesis, and is expressed in endothelial and lymphoid cells [32]. A role in the pancreatic β-cell has not been described previously. For SNP11, the T2DM-associated (G)_4 _was predicted to bind basic helix-loop-helix factor E47, whereas this binding was not present for the minor (G)_3 _allele. E47 is widely distributed, including pancreatic β-cells where it is well described as a regulator of insulin gene transcription [33]. Binding of E47 to the (G)_4 _allele might block activation by other transcription factors, and thus explain the association.

SNPs 1, 4, and 11 were not associated with T2DM in Caucasians. Thus, the association in African Americans may result from gene-gene or gene-environment interactions that are unique to this population. Alternatively, African Americans are known to be an admixed population, and concerns have been raised regarding spurious associations as a result of this admixture [34,35]. Several factors argue against a spurious association based on population structure, however. First, the 3 associated SNPs have similar frequencies in Caucasians and African Americans, such that population structure from admixutre would be less likely to lead to a spurious association. Second, of 87 SNPs previously typed, including 16 randomly chosen for large differences in African American and Caucasian allele frequencies and 71 chosen from candidate genes, only 3 have shown differences in allele frequencies that were significant at the p < 0.05 level. Thus, the findings in the current study have not been observed for multiple other genes tested.

Recently, lack of power has been raised as a reason for the inconsistent replication of associations [31,36,37], and very large sample sizes have been proposed to detect the small effects of variants such as the P12A polymorphism of the PPARγ gene or the E23K variant of the β-cell potassium channel, KCNJ11 [38]. By these standards, our study is small and likely would not have detected effects in either Caucasian or African American populations with a relative risk of below 1.4. However, among Caucasians we found no trend to an association. Indeed, for SNP 3, which showed the largest difference between cases and controls for any IPF1 SNP in Caucasians, achievement of 80% power to detect a difference significant at p < 0.05 would require 1700 cases and 1700 controls, based on a test of allelic association (3400 alleles for each group). Thus, although we cannot exclude an effect of these variants on T2DM risk that is comparable to that of PPARγ, the likelihood that a large enough study will be performed is small. The tagSNPs derived from our study will be useful should other investigators choose to undertake such a study.

The most intriguing of the unique African American variants is InsCCG243, which results in the inframe insertion of a proline in the carboxy-terminal polyproline tail, a region that is predicted to be involved in transactivation. We found this variant exclusively among African American subjects. We did not observe InsCCG243 among 142 Caucasian haplotypes, nor have other authors reported this variant in Caucasian populations [19,28]. InsCCG243 was reported previously in two French families, where it appeared to segregate in an autosomal dominant fashion and was associated with progressive insulin impairment [17]. The ethnic origin of these French families was not reported [17] and may have been of African or Afro-Caribbean. Expression of the InsCCG243 allele inhibited the endogenous IPF1 activation of the insulin gene by over 50% [17]. In the current study, InsCCG243 had an allele frequency of nearly 10% among diabetic subjects and 6.3% among controls. However, this difference did not reach statistical significance under any model in our studies. Hence, although InsCCG243 could contribute to 18% of T2DM among African Americans, the high prevalence observed in control individuals who had normal glucose tolerance and no family history of T2DM is inconsistent with the very high penetrance suggested by Hani et al [17]. Furthermore, among our African American families, InsCCG243 did not segregate in an autosomal dominant fashion (unpublished observations).

## Conclusion

We have carefully examined the *IPF1 *gene in two ethnic groups. We have extended earlier studies to the highly conserved upstream regulatory regions. Although we find no evidence for an association with T2DM among Caucasians, three putative regulatory variants are associated with T2DM in African Americans. Furthermore, a proline insertion in the transactivation domain was unique to African Americans and showed a trend to an association. These variants thus may explain part of the increased diabetes prevalence among African Americans. However, the lack of association of the same variants in Caucasians suggests gene-gene or gene-environment interactions, or perhaps a spurious association due to population structure. Additional population association and physiologic studies will be needed to confirm and extend these findings.

## Abbreviations

T2DM, type 2 diabetes

BMI, body mass index

IPF1, insulin promoter factor 1

## Competing interests

The author(s) declare that they have no competing interests.

## Authors' contributions

MAK was responsible for the direct conduct of the study including screening for sequence variation, assay design and data collection. XW assisted with genotyping, particularly of the InsCCG243 variant. TCH assisted with recruitment of all African American subjects and assisted in preparing data for analysis. SCE was responsible for study conception, oversight, all data analyses, and manuscript preparation. All authors read and approved the final manuscript.

## Acknowledgements

This work was supported by grants DK39311 and DK54636 from the National Institutes of Health/NIDDK, by the Research Service of the Department of Veterans Affairs, and by grants from the American Diabetes Association. Subject ascertainment, DNA preparation, data management and statistical assistance were supported in part by grant M01RR14288 from National Institutes of Health/National Center for Research Resources to the General Clinical Research Center (GCRC) of the University of Arkansas for Medical Sciences, College of Medicine. We thank the GCRC nursing staff for the support of this study, Judith Johnson Cooper for assistance with ascertainment of African American subjects in Arkansas, and Zhengxian Zhang for assistance with haplotype analyses in Arlequin.

## Pre-publication history

The pre-publication history for this paper can be accessed here:



## Figures and Tables

**Figure 3:**
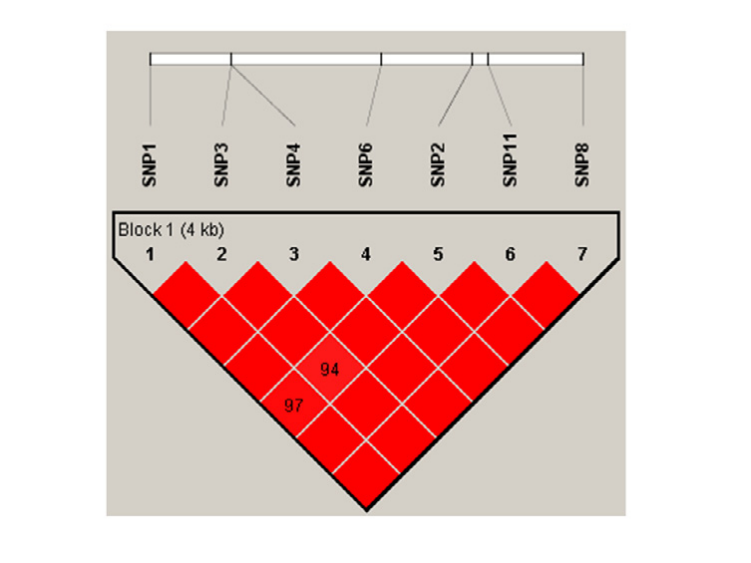
Linkage disequilibrium (D') plot of IPF1 gene in Caucasians. SNPs are shown by location as in Table 1 and Figure 1. As in Figure 2, plots were generated in HaploView v3.2. Squares without numbers represent D' values of 1.0; all numbers represent the D' value expressed as a percentile. Red squares represent pairs with LOD score for linkage disequilibrium of ≥ 2, blue squares represent D' = 1 but LOD<2, and white squares represent LOD<2 and D'<1.0. plots were generated using HaploView v3.2.

**Figure 4:**
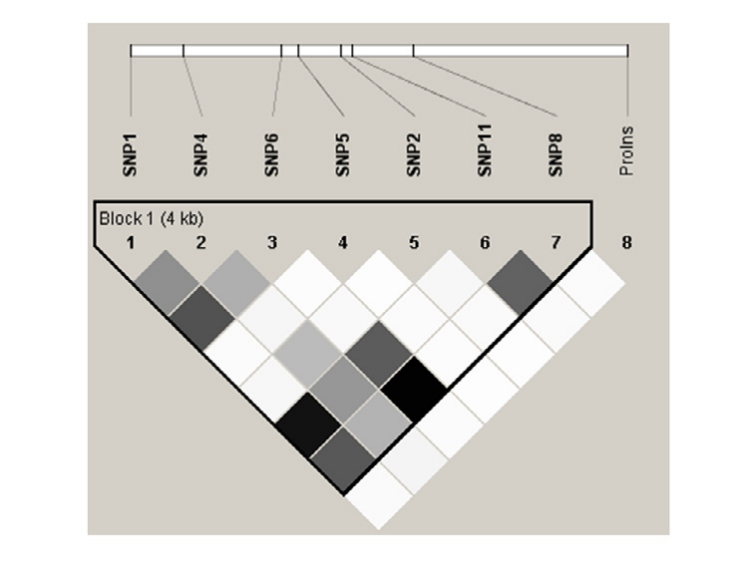
Linkage disequilibrium (r^2^) plot for in African Americans. Figure 4 is analogous to the D' plots shown in Figure 2. The r^2 ^value is shown on a grey scale, where white represents r^2 ^= 0, black represents r^2 ^= 1, and shades of grey represent 0<r^2^<1. All plots were generated using HaploView v3.2. SNP names and locations are as in Figure 2.

**Figure 5:**
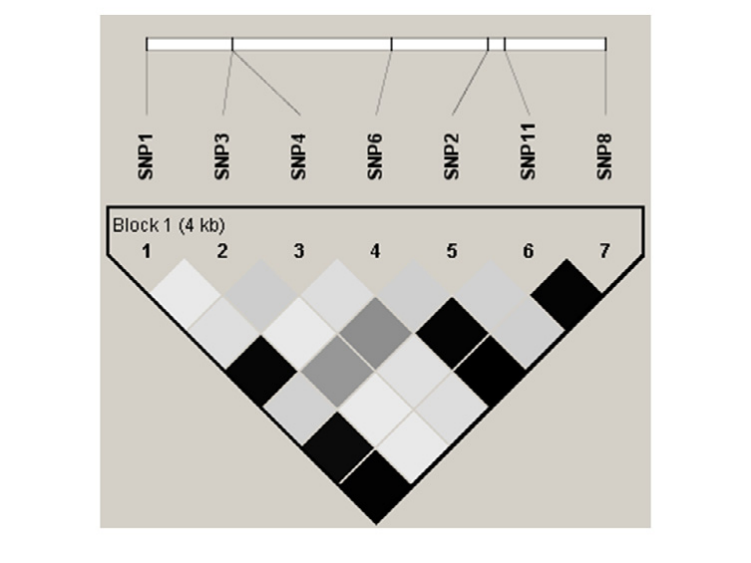
Linkage disequilibrium (r^2^) plot for Caucasians. Figure 5 is analogous to the D' plots shown in Figure 3. The value of r^2 ^is shown on a grey scale, where white represents r^2 ^= 0, black represents r^2 ^= 1, and shades of grey represent 0<r^2^<1. All plots were generated using HaploView v3.2. SNP names and locations are as in Figure 3.

**Table 1:** Primer sequences for IPF1 SNPs

**SNP NAME**	**FORWARD PRIMER 5' to 3'**	**REVERSE PRIMER 5' to 3'**	**SEQUENCE PRIMER 5' to 3'**	**Anneal Temp.**
SNP1	*ATTGCTTAGCCCTAGGAATAT	AGAGGGGCCAGGGAAACCCAG	GGATTGGAGAGAGGAAA	55°C
SNP2	GACGCCAGCTGCCCGTTCA	*CTGGCTGGCCGCACTAAGAG	AATTGGAACAAAAGCAG	55°C
SNP3 rs2293942	GGCAAGGACCTCCAGTATCAG	*CCCGAGCCATTTAACAG	CCTCCAGTATCAGCGAGGAC	55°C
SNP4 rs2293943	*GGCAAGGACCTCCAGTATCAG	CCCGAGCCATTTAACAG	TGAAAAAGTCGTTTATTAGC	55°C
SNP5 rs4002827	GATATCATGGAAAATGCAGCG	*GCTTCCCAATACAGCGAGG	GCAGAAGAGAGTGAGTGTT	55°C
SNP6	GTTTCGAGAAACGTCCTCATTT	*GCTTCTGGGGTCCTGACT	CAGTCAGAGGCTGGTCA	55°C
SNP8 rs4430606	Acttcccgcgcttcgtta	*CCAGCCCCTTCCTCTTTACT	CCAGGTAGGTGCAGAAAG	52°C
SNP11	GTCGTGCGGAGCTGTCAAAGCGAG	*CTGGAGCCGGGGATTT	AGCTGTCAAAGCGAGCAGGG	55°C
InsCCG243	CACGACGTTGTAAAACGACGAGACACATCAAGATCTGGTTCCAA	GGATAACAATTTCACACAGGGCAGCGGGCGGCACA		

* Denotes additional of universal primer sequence to the primer. Universal primer sequence is as follows: TCTGCTGCTCCGGTTCATAGATT-3'

**Table 2:** IFP1 Variation and Association with Type 2 Diabetes

**Name**	**Variat**	**dbSNP**	**Posit**	**Popul**	**Frequency, Caucasian**		**Frequency, Af. American**	
					
					**Cases**	**Controls**	**Cases**	**Controls**
SNP1	C/T	------	-3766	AA/Cauc	0.183 (0.143, 0.223)	0.192 (0.152, 0.232)	0.144 (0.118,0.170)	0.210^1 ^(0.167,0.253)
SNP3	T/C	rs2293942	-2890	Cauc	0.278 (0.232, 0.324)	0.249 (0.206, 0.292)	------	------
SNP4	A/T	rs2293943	-2877	AA/Cauc	0.339 (0.291, 0.387)	0.369 (0.321,0.417)	0.289 (0.255,0.323)	0.370^2 ^(0.321,0.419)
SNP6	G/T	------	-1263	AA/Cauc	0.196 (0.156, 0.236)	0.189 (0.151, 0.229)	0.119 (0.091,0.147)	0.129 (0.093, 0.165)
SNP5	C/T	rs4002827	-992	AA	------	------	0.071 (0.048, 0.094)	0.044 (0.021, 0.067)
SNP2	G/A	------	-279	AA/Cauc	0.458 (0.408, 0.508)	0.429 0.379, 0.479)	0.114 (0.086, 0.142)	0.139 (0.101, 0.177)
SNP11	(G)_4_/(G)_3_	------	-108	AA/Cauc	0.182 (0.143, 0.221)	0.177 (0.138, 0.216)	0.134 (0.108, 0.160)	0.217^3 ^(0.175, 0.259)
SNP8	G/T	rs4430606	+918	AA/Cauc	0.197 (0.157, 0.237)	0.191 (0.152, 0.232)	0.115 (0.087, 0.143)	0.133 (0.096, 0.170)
InsCCG243	InsCCG	------	+4437	AA	-----	-----	0.091 (0.069, 0.113)	0.060^4 ^(0.035, 0.085)

Name, name from Figure 1 and text; variant, major/minor allele except for InsCCG243, in which the insertion is the minor allele; dbSNP, catalog number in public database if available; position, location relative to ATG start; Populat, population in which variant was detected, AA is African American, Cauc is Caucasian; frequency, minor allele frequency. Frequencies are shown with 95% confidence intervals in parentheses. Significance by Fisher Exact test: ^1^p = 0.007;^2^p = 0.008; ^3^p = 0.0008; ^4^p = 0.088; simulated p values based on 10,000 replicates were 0.027, 0.029, 0.002, and 0.28, for SNP1, SNP4, SNP11, and InsCCG243, respectively. No other simulated p values approached significance.

**Table 3:** Raw Counts for Caucasian and African American Case Control Studies

**SNP Name**	**Caucasian**						**African American**					
	**Major/Major**		**Major/Minor**		**Minor/Minor**		**Major/Major**		**Major/Minor**		**Minor/Minor**	

	**DM**	**Cnt**	**DM**	**CNT**	**DM**	**CNT**	**DM**	**CNT**	**DM**	**CNT**	**DM**	**CNT**

**SNP 1**	120	124	54	59	6	7	249	117	84	60	7	9
**SNP 2**	54	61	97	95	38	34	197	124	53	35	2	3
**SNP 3**	97	73	15	108	71	12	---	---	---	---	---	---
**SNP 4**	84	82	43	76	89	26	169	147	25	79	75	31
**SNP 5**	---	---	---	---	---	---	210	143	34	14	1	0
**SNP 6**	124	125	56	58	7	9	200	119	53	43	2	3
**SNP 8**	123	123	56	58	9	7	198	115	50	41	2	3
**SNP 11**	125	8	51	125	53	6	253	114	76	57	7	11
**ProIns**	---	---	---	---	---	---	265	57	1	155	21	0

Numbers of individuals with each genotype are shown for the SNPs in Table 1. Significance by allelic association is shown in Table 1 with confidence intervals for allele frequencies. Note that allelic association was the primary test performed. Data not shown (--) was not typed in the full case control set because of low frequency. Counts differ slightly due to genotypes that were not called, and because additional African American samples were typed for SNPs 1, 4, 11, and ProIns (proline insertion) based on initial data showing an association.

**Table 4:** Haplotypes observed in the Caucasian Population

**Haplotype**	**Case Freq**	**Control Freq**	**p value**
CTAGGIG	0.337	0.364	0.4406
CCTGAIG	0.269	0.247	0.4939
TTTTGDT	0.192	0.187	0.8625
CTTGAIG	0.184	0.186	0.936

Haplotypes observed at over 1% frequency are shown for SNPs observed in the Caucasian population (SNPs 1, 3, 4, 6, 2, 11, and 8). SNP 11 is shown as I (insertion, G_4 _or 4 G's) or D (deletion, G_3 _or 3 G's). All other SNPs are shown as listed in Table 2.

**Table 5:** Haplotypes for 8, 4, and 3 Markers Observed in the African American Population

Variants	No	Haplotype	Case Freq	Control Freq	ChiSq	P value	Simulated p value	Global p
1,4,6,5,2,11,8,P								
	1	CAGCGIGD	0.531	0.511	0.326	0.5683	1	0.01
	2	TTTCGDTD	0.103	0.145	3.336	0.0678	0.465	
	3	CTGCAIGD	0.112	0.124	0.247	0.6193	1	
	4	CAGCGIGI	0.108	0.064	4.676	0.0306	0.239	
	5	CAGTGIGD	0.072	0.046	2.389	0.1222	0.693	
	6	TTGCGDGD	0.031	0.067	5.873	0.0154	0.131	
	7	CTGCGIGD	0.026	0.024	0.014	0.9057	1	
1,4,11,P								
	1	CAID	0.622	0.565	3.197	0.0738	0.3053	0.01
	2	TTDD	0.138	0.209	9.084	0.0026	0.0094	
	3	CTID	0.143	0.151	0.134	0.714	0.9995	
	4	CAII	0.088	0.059	2.735	0.098	0.3809	
1,4,11								
	1	CAI	0.71	0.625	7.96	0.0048	0.02	0.01
	2	TTD	0.138	0.209	9.085	0.0026	0.011	
	3	CTI	0.145	0.152	0.091	0.763	1	

Haplotypes observed at over 1% frequency for all 8 variants, the 4 variants typed in additional individuals, and the three variants that showed an association with T2DM. Simulated p values are based on 10,000 simulations in HaploView 3.2; global p values are based on simulations conducted in Phase 2.1.1.
